# Impact of Weight Status on Hemodynamic Parameters and Aerobic Fitness in School-Aged Children: A Study in a Rural School Community

**DOI:** 10.3390/ijerph21101340

**Published:** 2024-10-10

**Authors:** Ncomi Primrose Lukhele, Lourens Millard, Gerrit Jan Breukelman

**Affiliations:** Department of Human Movement Science, Faculty of Science and Agriculture, University of Zululand, KwaDlangezwa 3886, South Africa; ncomikhinti6@gmail.com (N.P.L.); breukelmang@unizulu.ac.za (G.J.B.)

**Keywords:** aerobic fitness, hemodynamic factors, children, obesity, body composition, weight

## Abstract

Maintaining a high level of physical activity provides significant health benefits for children, particularly in enhancing cardiorespiratory fitness and maintaining a healthy weight. This study aimed to investigate the impact of weight status on children’s hemodynamic parameters and aerobic fitness levels. This cross-sectional study included 350 rural primary school children (146 boys and 204 girls) aged 11–13 years, randomly selected from two schools in the King Cetshwayo District, KwaDlangezwa area of KwaZulu-Natal. The anthropometric measurements recorded included height, weight, waist circumference, and hip circumference, with percentiles calculated using Cole’s Lambda, Mu, and Sigma method. Additionally, skinfold measurements were obtained at four sites (biceps, triceps, subscapular, and suprailiac). Hemodynamic measurements included systolic blood pressure, diastolic blood pressure, and heart rate. Aerobic fitness was assessed using the 20 m shuttle run test, which evaluated speed, level, and age. The healthy group demonstrated significantly lower ratios in several measurements compared to those of the overweight group, i.e., the VO_2_max was 6% higher (<0.001), the waist-to-hip ratio was 6% lower (<0.001), the body fat percentage was 16% lower (<0.001), the waist circumference was 12% lower (<0.001), and the resting heart rate was 3% lower (*p* ≤ 0.055). Differences in systolic and diastolic blood pressure were also observed, with the systolic pressure 2% lower (*p* ≤ 0.116) and the diastolic pressure 3% lower (*p* ≤ 0.086) in the healthy group. The study revealed a significant association between body weight status, aerobic fitness, and blood pressure parameters. Conducted in rural primary schools in KwaDlangezwa, KwaZulu-Natal, the research highlights notable correlations among weight status, aerobic competence, and blood pressure (BP) in children aged 11 to 13 years. The findings indicate that children with a healthy body weight showed higher VO_2_max levels and a reduced risk of developing childhood obesity and hypertension, unlike their overweight or obese peers, who displayed lower aerobic fitness and higher blood pressure.

## 1. Introduction

Childhood obesity and O/W are conditions marked by an excessive or abnormal accumulation of fat, leading to various health problems in children, such as diabetes, heart disease, osteoporosis, and high blood pressure (HBP) [1]. Body mass index (BMI), calculated by dividing weight by height in meters squared, is a useful metric for estimating children’s weight status [2,3]. The World Health Organization (WHO) recommends using BMI cut-off points to categorize adult weight status into four groups: underweight (BMI < 18.5), normal weight (BMI < 25), overweight (BMI < 30), and obese (BMI ≥ 30) [4,5]. For children, BMI is used to determine weight categories based on age and gender, but there are no universal guidelines for classifying weight in children [2,6].

The Centers for Disease Control and Prevention (CDC) growth charts provide a visual representation of a child’s physical development, showing the distribution of specific body measurements in children aged 2 to 18 years [6]. Childhood weight is classified into three categories: underweight, overweight, and obese [3，^6^]. A BMI above the 95th percentile indicates obesity, while one between the 85th and 95th percentiles signals a risk of overweight. A BMI below the 5th percentile is categorized as underweight [6].

The concerning trend of escalating childhood obesity rates continues to pose significant health hazards among children [7]. Previous studies [7,8,9] have underscored the critical role of aerobic fitness, defined as the body’s ability to efficiently supply oxygen during physical exertion, in promoting overall health, especially among younger children [9,10]. Encouraging children to participate in aerobic fitness activities not only improves their performance but also their body weight and hemodynamic parameters, which refer to the forces and dynamics of blood flow within the cardiovascular system, and these include components such as BP, vascular resistance, cardiac output (CO), stroke volume (SV), heart rate (HR), venous return, blood volume (BV), blood viscosity, vascular compliance, etc. However, this study primarily focused on systolic blood pressure (SBP), which is the pressure in the arteries when the heart contracts and pumps blood, while diastolic blood pressure (DBP) indicates the pressure in the arteries when the heart is at rest between beats, and HR refers to the number of times the heart beats per minute. Maintaining healthy hemodynamic parameters is crucial, as it helps lower the risk of developing chronic diseases later in life [7,10,11]. Additionally, studies show that children who are overweight (O/W) or obese are more likely to develop high blood pressure (HBP) at an earlier age and tend to have lower levels of aerobic fitness compared to their peers exhibiting a healthy weight [12,13]. Thus, it is vital for children to maintain an age-appropriate healthy weight.

The primary concern regarding childhood obesity is that approximately 50% of obese children grow up to be obese adults, with 70% of these adults being obese after the age of thirty. Thus, measures to lessen and prevent childhood obesity are necessary [12,13]. Furthermore, children who are O/W have a higher chance of dying as adults due to metabolic and cardiovascular diseases [14,15]. According to Tuan et al. [16], there is evidence that obese children exhibit decreased aerobic fitness levels during childhood, and this trend continues for more than a decade [17,18]. Only about one-third of South African primary school children engage in the recommended levels of physical activity, particularly in rural schools [3]. This highlights the clear connection between poor levels of aerobic fitness and childhood obesity [17].

Childhood overweight and obesity are well-studied topics globally [12,19]. However, childhood obesity rates continue to rise unabated and are common in developing countries [20,21]. Little research has been conducted in South Africa regarding the prevalence of obesity and overweight in connection to hemodynamic variables and aerobic fitness [3], especially among African children living in rural areas. No published information exists regarding how the weight status of KwaDlangezwa rural school children affects their levels of aerobic fitness and hemodynamic parameters. Therefore, this research aims to fill a knowledge gap by examining the impact of weight status on hemodynamic variables and aerobic fitness levels in school-aged children in the KwaDlangezwa area of KwaZulu-Natal.

## 2. Materials and Methods

### 2.1. Participants and Procedures

This cross-sectional study recruited 350 participants (146 boys and 204 girls) from two primary schools situated in the KwaDlangezwa region. The sampled population comprised children aged 11 to 13 years, encompassing both genders. Using an online sample-size calculator (Raosoft^®^) http://www.raosoft.com/samplesize.html (accessed on 14 July 2024). (Raosoft, Inc. Seattle, WA, USA). The study employed the parameters of a 5% error margin and a 95% confidence interval to ascertain the requisite sample size. Given the combined population of 3800 children within Khandisa Primary School and Matshangule Primary School, the algorithmic calculation determined that a sample of 350 children would be sufficient for the study’s objectives.

Participants in the study were excluded if they were taking any medications, had a history of cardiac or neuromuscular diseases or events, or had experienced physical activity limitations in the past six months [22]. Unlike children who did not participate in organized sports, children who did were instructed to refrain from attending the training sessions and were advised to get adequate sleep for at least two days before the testing sessions. Participants were instructed to eat a small meal at least three hours before the VO_2_max test and to stay properly hydrated both before and throughout the tests [11,23,24].

Research measurements were conducted in classrooms and on the sports fields of two elementary schools, encompassing a range of anthropometric traits including height, weight, waist circumference, and hip circumference. Additionally, four-site skinfold measurements were taken at the biceps, triceps, subscapular, and suprailiac regions. Hemodynamic indicators such as systolic blood pressure (SBP), diastolic blood pressure (DBP), and heart rate (HR) were also recorded. Aerobic fitness was assessed using the 20 m shuttle run test, evaluating speed, level, and age-related metrics.

The study procedure is summarized with the diagram below.

### 2.2. Inclusion Criteria

**Figure 1:**
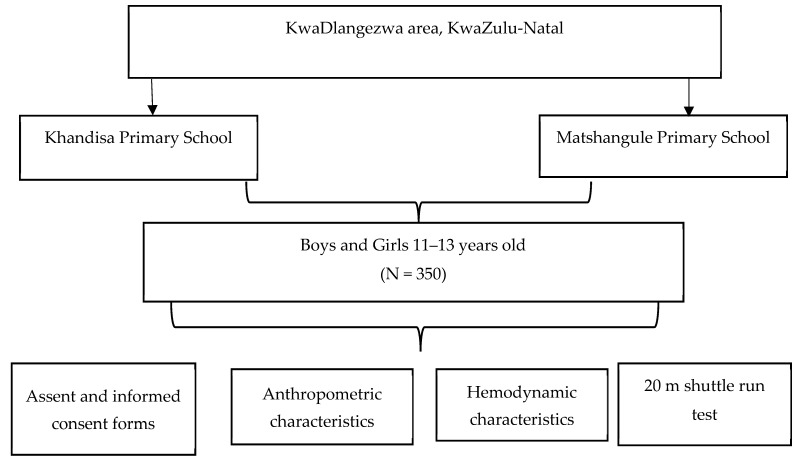
Procedures of the study.

To participate in the study, individuals needed to be currently enrolled as active learners at specified elementary schools in KwaDlangezwa, KwaZulu-Natal. Eligible participants fell within the age range of 11 to 13 years old and were in grades 5 to 7, as depicted in Figure 1. This phase of childhood development is characterized by hormonal changes [25], irregular eating habits, and increased levels of physical inactivity, all of which, in previous research, have been linked to the onset of obesity [26,27]. Targeting this age group was therefore crucial for the objectives of the study.

### 2.3. Exclusion Criteria

Children outside the age range of 11 to 13 years old were not included in the study. Exclusion criteria also applied to those with contraindications to exercise, taking medication, having a history of neuromuscular or cardiac conditions or injuries, experiencing recent physical activity limitations, or showing signs of anxiety or distress during testing [28,29]. Additionally, participants who had consumed caffeinated beverages prior to testing were also excluded from the study [30,31].

### 2.4. Data Collection Process

Data collection occurred in three phases over a span of three weeks. Testing took place each weekday from Monday to Friday and was conducted between 8 a.m. and 1 p.m. Anthropometric measurements, including height, weight, waist circumference, hip circumference, and skinfold measurements at four sites (biceps, triceps, subscapular, and suprailiac), were conducted in each subject’s classroom. Prior to the 20 m shuttle run test, hemodynamic measurements, such as heart rate and systolic and diastolic blood pressure, were taken in the mornings within various school classrooms. During the shuttle run test, children were grouped by gender, with each group consisting of eight to ten participants, and the test was administered on the school grounds. All measurements were closely supervised by the researcher and research assistants throughout the process.

#### Anthropometric Measurements

- Height and Weight

The participants’ height and weight were measured according to the protocol established by the International Society for the Advancement of Kinanthropometry (ISAK) [32]. Participants stood barefoot against a stadiometer in order to measure height accurately to the nearest 0.1 cm. For weight measurement, participants were lightly dressed in underwear and a T-shirt and stood on a digital weighing scale (Tanita HD 309^®^, Creative Products, MI, USA Holland, Michigan), which was calibrated every 15 measurements to ensure that readings were accurate to the nearest 0.1 kg.

To calculate body mass index (BMI), the CDC NHS online BMI calculator for children and teens was utilized. BMI was calculated by dividing weight in kilograms by height squared (kg/m^2^). BMI classifications (normal, underweight, overweight, obese) were determined using CDC reference percentiles (the 5th, 10th, 15th, 25th, 50th, 75th, 85th, 90th, and 95th percentiles) for BMI adjusted for age and sex [6,31]. These percentiles were derived from anthropometric reference data for children and adults provided by the CDC for the years 2015–2018.

b. Waist to Hip (WHR)

The participants’ waist and hip circumferences were measured while they faced forward with arms relaxed at their sides. A non-distendible measuring tape (Holtain Ltd. Crymych, Pembrokeshire, Wales, United Kingdom) was used to measure waist circumference at the narrowest point between the lower costal border and the iliac crest and hip circumference at the widest part of the hip [19]. Waist–hip ratio (WHR) was calculated using the following formula: waist circumference ÷ hip circumference, following guidelines from Ref. [19]. To categorize participants, the 3rd, 10th, 25th, 50th, 75th, 90th, and 97th percentiles for age and gender were utilized. This classification system helped differentiate children into low, medium, or high-risk categories based on their waist-to-hip ratio [19,33].

c. Skinfolds

A calibrated skinfold caliper (Lange, Beta Technology Inc., Cambridge, MD, USA) was used to measure the skinfold thickness at four sites (triceps, biceps, subscapular, and suprailiac) on the right side of the body. Two measurements were taken at each site, and if the difference between the measurements exceeded 2 mm, a third measurement was taken for accuracy. The mean value of the two closest measurements was then calculated, and the sum of these four skinfold measurements (mm) was used for further analysis. Finally, the percentage of body fat (%BF) was calculated by adding the triceps and subscapular skinfolds (∑TS) using Slaughter et al.’s method [34]. This equation is widely acceptable for use with children of diverse ethnicities. For boys (all ages), %BF = 1.2 (∑TS) − 0.008 (∑TS)2 − 3.2. For girls (all ages), %BF = 1.33 (∑TS) − 0.013 (∑TS)2 − 2.5, where ∑TS is the sum of the triceps and subscapular skinfolds [30,35].

d. Hemodynamic Measurements

Before performing the 20 m shuttle run test (20-mSRT), the participants’ systolic blood pressure (SBP), diastolic blood pressure (DBP), and heart rate (HR) were assessed [7]. These measurements were taken using the Microlife BP A2 Basic automated brachial sphygmomanometer, as described by Bing et al. [36], using a child-sized cuff designed for arm circumferences of 17–22 cm. The participants were asked to remove their outer clothing and sit comfortably for at least 10 min prior to measurement. The cuff was placed on the left arm at the level of the brachial artery, following the guidelines set by Cohen et al. [37]. For both SBP and DBP, two readings were taken, with the lower value being recorded. HR was measured in beats per minute (bpm) using a pulse oximeter placed on the participant’s index finger, with the fingernail facing upwards. To ensure accuracy, participants were instructed to remain still during the measurement. The average of the HR measurements was used for further analysis, following the procedure outlined by Silva et al. [38]. Throughout the process, participants maintained a consistent seated position, with their arms at heart level. Blood pressure readings were categorized based on American Academy of Pediatrics guidelines, considering variations in BP by age and gender, Therefore, similar to the task force classification, the blood pressure (BP) categories were defined as follows: low BP (systolic BP (SBP) and diastolic BP (DBP) below the 5th percentile), normal BP (systolic BP (SBP) and diastolic BP (DBP) below the 90th percentile), and hypertension (SBP or DBP at or above the 95th percentile), as detailed by Flynn et al. [39]. The results were classified into “low”, “normal”, and “high” categories for both boys and girls, taking into account age and height criteria. Heart rate classifications were based on standards established by Fleming et al. [40].

e. Aerobic Fitness Test (20 m Shuttle Run Test)

Aerobic fitness was evaluated using the 20 m shuttle run test (20-mSRT), also known as the beep test, a well-established and validated methodology for assessing children’s aerobic capacity. The prediction of aerobic capacity based on test results is accurate for children of all weight categories, including those who are overweight, obese, or of normal weight [41]. Based on the framework by Leger et al. [42], this test involved continuous running between two points 20 m apart, with auditory cues signaling intervals marked by a “beep” sound. Participants started at 8.5 km/h, and the speed increased by 0.5 km/h every minute using the beep shuttle run advanced VER0320 program [43]. Test administration followed established guidelines, including a familiarization trial for participants. Termination criteria included voluntary withdrawal due to fatigue, failure to meet the designated marker within the allotted time, inability to reach the finish lines in sync with the audio signals on two consecutive attempts, or completion of all predefined speed levels [44]. Performance was quantified by the number of shuttles completed, with the final running speed (km/h) achieved at the last completed stage used as an indicator of individual performance [44]. The data analysis utilized standard protocols within the assessment software, calculating maximum oxygen uptake (VO_2_max) indirectly, using the following equation: VO_2_max (ml/kg/min) = 31.025 + (3.238 × speed) − (3.248 × age) + (0.1536 × speed × age). The scores were then categorized into fitness levels, as follows: very poor (<10%), poor (10–25%), fair (25–75%), good (75–90%), and excellent (≥90%) [44].

f. Data Analysis

In this quantitative study, anthropometric characteristics, including weight, height, waist circumference, hip circumference, and skinfold measurements, were treated as independent variables. The dependent variables for this analysis were the VO_2_max and hemodynamic parameters (systolic blood pressure, diastolic blood pressure, and heart rate).

Data analysis was performed using the Statistical Package for Social Sciences (SPSS), version 22 (IBM Corporation, Armonk, NY, USA). The descriptive statistics were calculated for the collected data, which included means, standard deviations, percentage differences, and ranges.

Due to the non-normal distribution of the dependent variable (VO_2_max), the Mann–Whitney U test was utilized to evaluate differences between independent groups, specifically comparing healthy and overweight participants.

This methodological approach was designed to highlight the relative importance of various health-related fitness components and to confirm the statistical significance of the observed differences. A significance level of *p* ≤ 0.05 was established to assess the robustness of the findings.

## 3. Results

Among the 350 children studied, 57% were classified as healthy, while 43% were identified as overweight. Children were categorized based on specific criteria, as follows: children with waist circumference values below the 85th percentile for age and gender were considered healthy, whereas those with values at or above the 90th percentile were classified as overweight [45]. For the waist-to-hip ratio, a healthy classification was given to girls with a ratio of less than 0.85 and to boys with a ratio of less than 0.90; children with ratios equal to or greater than these thresholds were deemed overweight [19]. Additionally, body fat percentages below the 75th percentile for girls and below the 50th percentile for boys were considered healthy, while percentages meeting or exceeding these thresholds were classified as overweight [46]. VO_2_max cut-off values for classification were set at 42 mL·kg^−1^min^−1^ for boys and 35 mL·kg^−1^min^−1^ for girls [47]. Analysis revealed that the VO_2_max values in the overweight group were significantly lower than those in the healthy group. Furthermore, regarding age, the healthy group had a mean ± SD of 11.99 ± 0.77 years, while the overweight group had a mean ± SD of 12.04 ± 0.76 years.

### 3.1. Anthropometric Characteristics

The anthropometric characteristics of the 350 children studied are summarized in Table 1. Participants were classified into two groups: healthy (57%) and overweight (43%). Classification criteria included waist circumference, waist-to-hip ratio, body fat percentage, and VO_2_max values.

The results show significant differences between healthy and overweight groups across various anthropometric measures, with the overweight group exhibiting higher values for weight, waist circumference, hip circumference, waist-to-hip ratio, and body fat percentage (all *p* < 0.001) (Table 1).

### 3.2. Hemodynamic Characteristics

Although differences in resting heart rate and systolic and diastolic blood pressure were observed between the groups, none reached statistical significance (*p* > 0.05) (Table 2).

### 3.3. Aerobic Characteristics

The healthy group displayed significantly higher aerobic capacity, indicated by VO_2_max values, beep speed, and beep level, compared to that of the overweight group, all with *p*-values less than 0.001 (Table 3).

Overall, the analysis indicates that children in the healthy group exhibited superior anthropometric, hemodynamic, and aerobic characteristics compared to those classified as overweight. Significant differences were noted in key measures such as weight, body fat percentage, and VO_2_max, underscoring the importance of maintaining a healthy lifestyle in this population.

## 4. Discussion

This study aimed to examine the impact of body weight on aerobic fitness and hemodynamic variables in rural primary school children aged 11 to 13 years. Initially, thirteen variables were considered, with a particular focus on age, resting heart rate (RHR), systolic blood pressure (SBP), diastolic blood pressure (DBP), waist circumference (WC), waist-to-hip ratio (WHR), body fat percentage (FAT%), and VO_2_max across genders. The cohort displayed a notable prevalence of overweight and obesity, as indicated by FAT% (29%), WHR (6%), and central obesity, as assessed by WC (12%) (Table 1). These findings align with the results of previous research by He et al. and Lopez-Gil et al. [48,49], which identified associations between aerobic fitness and various anthropometric measurements. Furthermore, as noted by Pojskic and Eslami [11], the healthy group exhibited a 6% higher VO_2_max (Table 3) compared to that of their overweight counterparts, underscoring the adverse effects of poor body weight on aerobic fitness levels.

Additionally, a study by Brand et al. [50] revealed significant correlations between WC, FAT%, and aerobic fitness in overweight/obese children, a pattern not observed in those of normal weight. Further analysis in our study confirmed significant associations between aerobic fitness and both WC and FAT%. Children with a healthier weight status, which were characterized by smaller WC, lower FAT%, and WHR values, demonstrated superior aerobic fitness levels. However, findings by Zadarko-Domaradzka [51] indicated that among children aged 10 to 15 years, WHR was the least sensitive predictor of cardiorespiratory fitness (CRF) (R^2^ = 39.2%). This suggests that maintaining an ideal body weight plays a crucial role in reducing the risk of developing metabolic diseases in the future.

In this study, a significant 6% difference in waist circumference was observed between the healthy and overweight groups. WC serves as a measure of central obesity and total body fat percentage [48,50]. Therefore, Zadarko-Domaradzka [51] revealed that schoolchildren with larger waist circumferences were more likely to have their VO_2_max levels classified below the P60–P80 percentile healthy range, which supports our findings. The observed 6% difference in WC is consistent with the results reported by Brand et al. [50]. Notably, only 57% of the population falls within the healthy waist circumference parameters, indicating that a significant portion of this population may be at risk for central obesity in the future.

Regarding the hemodynamic variables, the results for systolic blood pressure (SBP) indicated a relationship between healthy WHR and WC when comparing results for the healthy group with those for the at-risk group, consistent with findings by Burgos et al. [52]. Conversely, Sarni et al. [53] found no correlation between WC and either SBP or DBP in a sample of 65 preschoolers from low socioeconomic backgrounds. In contrast, a high FAT% was linked to increased blood pressure readings in the overweight group, supporting studies that suggest associations between obesity measures and elevated blood pressure [44,53]. Additionally, Cauduro et al. [54] reported that overweight/obese children who adhered to recommended physical activity guidelines were less likely to experience high blood pressure levels, regardless of sex, age, or socioeconomic status.

In our study, no significant correlation was found between resting heart rate (RHR) and WHR values, which contrasts with the findings of Itagi et al. [55]. Their research showed that higher resting heart rates were positively associated with obesity indices in obese individuals compared to those who were not obese. This stipulates the role of resting heart rate as a key indicator of cardiovascular health and metabolic function. Overall, the study highlights the importance of aerobic fitness and body weight as modifiable factors for the early prevention of hypertension in children [56]. These findings carry important implications for public health interventions aimed at rural school settings. Early identification of elevated body fatness and low aerobic fitness can inform strategies designed to promote physical activity and health education among children and their communities. Such interventions hold the potential to mitigate future risks of cardiovascular diseases and obesity-related complications [57].

## 5. Limitations

The study’s sample size featured a notable gender disparity, with 146 boys and 204 girls participating, which may impact the generalizability of the results to the broader population. This gender difference underscores the importance of considering potential gender-specific differences in body weight and cardiovascular health outcomes among children. The inclusion of a voluntary participation criterion in the study potentially reduced its ecological validity. Motivating ongoing participation and adherence to study protocols can be challenging in voluntary studies, affecting the consistency and reliability of data collection over time. The use of three indicators for body composition, WC, WHR, and FAT%, was time-consuming and may have introduced variability in measurements. Factors such as unpredictable high temperatures during testing sessions could have influenced these parameters, affecting the accuracy and reliability of the results. During the study, children experienced discomfort from heart rate monitors (pulse oximeters) on their fingers, leading some to touch remove them, potentially compromising the validity of heart rate measurements. Finally, the study also lacked the ability to control sleep. This issue highlights the practical challenges of conducting physiological measurements in children, particularly in field settings.

To ensure participant safety and maintain data integrity in line with ACSM guidelines [58], children with contraindications to exercise testing (e.g., fever, injuries, elevated resting heart rate, or blood pressure) were excluded. Additionally, participants showing signs of anxiety or distress during testing were not compelled to continue, which could have affected the sample’s representativeness and the study’s outcomes. It is noteworthy that the study opted not to use BMI as a predictor of childhood obesity, focusing instead on more robust indicators like WHR, WC, and FAT%. These indicators are considered more accurate in assessing O/W and obesity, central adiposity, and metabolic health risks in children [57,59].

## 6. Conclusions

This study conducted in rural primary schools in KwaDlangezwa, KwaZulu-Natal, identified significant correlations between weight status, aerobic fitness, and hemodynamic parameters in children aged 11 to 13 years. The results indicated that children with a healthy body weight demonstrated higher VO_2_max levels and are at a low risk of developing obesity and hypertension compared to their overweight/obese counterparts, who exhibited lower aerobic fitness and elevated blood pressure levels.

These findings elaborate the critical importance of maintaining a healthy body weight from an early age to mitigate the risk of chronic diseases later in life. Furthermore, this research contributes significantly to the limited body of literature on childhood obesity in South Africa, particularly within rural contexts. By identifying specific body weight metrics associated with adverse health outcomes, the study provides valuable insights for formulating targeted interventions aimed at increasing physical activity and improving health education in schools.

Future research should aim to explore these relationships across more diverse populations, with a focus on gender differences, to enhance our understanding and inform public health strategies. Ultimately, effectively addressing childhood obesity necessitates comprehensive efforts that integrate physical activity promotion, health education, and community engagement, thereby fostering healthier lifestyles from a young age.

## Figures and Tables

**Table 1:** Anthropometric characteristics of healthy weight and overweight children.

Variable	Healthy Weight (*n* = 201)	Overweight (*n* = 149)	Difference (%)	Significance (*p*-Value)
Age	11.99 ± 0.77	12.04 ± 0.76	0.4	0.546
Height (m)	148.36 ± 8.28	149.29 ± 8.08	0.6	0.293
Weight (kg)	41.02 ± 7.71	49.92 ± 12.87	18	<0.001 *
Waist circumference (cm)	62.59 ± 5.17	70.85 ± 10.05	12	<0.001 *
Hip circumference (cm)	82.08 ± 7.08	87.42 ± 10.82	6	<0.001 *
Waist–hip ratio	0.76 ± 0.04	0.81 ± 0.06	6	<0.001 *
Body fat	20.72 ± 4.46	24.99 ± 5.16	16	<0.001 *

Mean ± SD; *: statistically significant (*p* ≤ 0.05).

**Table 2:** Hemodynamic characteristics of healthy weight and overweight children.

Variable	Healthy Weight (*n* = 201)	Overweight (*n* = 149)	Difference (%)	Significance (*p*-Value)
Resting heart rate (bpm)	83.92 ± 13.45	86.92 ± 11.73	3	0.055
Systolic blood pressure (mmHg)	113.85 ± 11.11	115.81 ± 12.14	2	0.116
Diastolic blood pressure (mmHg)	71.32 ± 13.57	73.79 ± 12.96	3	0.08

**Table 3:** Aerobic characteristics of healthy weight and overweight children.

Variable	Healthy Weight (*n* = 201)	Overweight (*n* = 149)	Difference (%)	Significance (*p*-Value)
Beep speed (km/h)	9.07 ± 0.50	8.67 ± 0.55	4	<0.001 *
Beep level	3.81 ± 1.04	3.05 ± 1.15	20	<0.001 *
VO_2_max	38.26 ± 3.09	36.07 ± 313	6	<0.001 *

Mean ± SD; *: statistically significant (*p* ≤ 0.05).

## Data Availability

The data supporting the study findings are available from the corresponding author upon request.
